# Neuregulin Facilitates Nerve Regeneration by Speeding Schwann Cell Migration via ErbB2/3-Dependent FAK Pathway

**DOI:** 10.1371/journal.pone.0053444

**Published:** 2013-01-02

**Authors:** Hung-Ming Chang, Ming-Kwang Shyu, Guo-Fang Tseng, Chiung-Hui Liu, Hung-Shuo Chang, Chyn-Tair Lan, Wen-Ming Hsu, Wen-Chieh Liao

**Affiliations:** 1 Department of Anatomy, School of Medicine, College of Medicine, Taipei Medical University, Taipei, Taiwan; 2 Department of Obstetrics and Gynecology, National Taiwan University Hospital, Taipei, Taiwan; 3 Department of Anatomy, College of Medicine, Tzu Chi University, Hualien, Taiwan; 4 Graduate Institute of Anatomy and Cell Biology, College of Medicine, National Taiwan University, Taipei, Taiwan; 5 Department of Anatomy, Faculty of Medicine, Chung Shan Medical University, Taichung, Taiwan; 6 Division of Pediatric Surgery, Department of Surgery, National Taiwan University Hospital, Taipei, Taiwan; 7 Department of Pediatrics, Chung Shan Medical University Hospital, Taichung, Taiwan; University of Nebraska Medical Center, United States of America

## Abstract

**Background:**

Adequate migration of Schwann cells (Sc) is crucial for axon-guidance in the regenerative process after peripheral nerve injury (PNI). Considering neuregulin-erbB-FAK signaling is an essential pathway participating in the regulation of Sc migration during development, the present study is aimed to examine whether neuregulin would exert its beneficial effects on adult following PNI and further determine the potential changes of downstream pathway engaged in neuro-regeneration by both *in vitro* and *in vivo* approaches.

**Methodology and Principal Findings:**

Cultured RSC96 cells treated with neuregulin were processed for erbB2/3 immunofluorescence and FAK immunoblotings. The potential effects of neuregulin on Sc were assessed by cell adherence, spreading, and migration assays. In order to evaluate the functional significance of neuregulin on neuro-regeneration, the *in vivo* model of PNI was performed by chronic end-to-side neurorrhaphy (ESN). *In vitro* studies indicated that after neuregulin incubation, erbB2/3 were not only expressed in cell membranes, but also distributed throughout the cytoplasm and nucleus of RSC96 cells. Activation of erbB2/3 was positively correlated with FAK phosphorylation. Neuregulin also increases Sc adherence, spreading, and migration by 127.2±5.0%, 336.8±3.0%, and 80.0±5.7%, respectively. As for *in vivo* study, neuregulin significantly accelerates the speed of Sc migration and increases Sc expression in the distal stump of injured nerves. Retrograde labeling and compound muscle action potential recordings (CMAP) also showed that neuregulin successfully facilitates nerve regeneration by eliciting noticeably larger CMAP and promoting quick re-innervation of target muscles.

**Conclusions:**

As neuregulin successfully improves axo-glial interaction by speeding Sc migration via the erbB2/3-FAK pathway, therapeutic use of neuregulin may thus serve as a promising strategy to facilitate the progress of nerve regeneration after PNI.

## Introduction

Peripheral nerve injury (PNI) is one of the most common and important injuries in the current societies [1]–[3]. Previous studies had indicated that after PNI, complete fragmentation of distal axons, degradation of myelin sheath and infiltration of macrophages would occur in the distal stump of lesioned nerves [4], [5]. Biochemical reports also demonstrated that following PNI, remnant Schwann cells (Sc) would gradually migrate to the injured site and provide supportive effects to proximal axons which promotes successive neuro-regeneration [6], [7]. By expressing a variety of trophic factors, Sc could serve as a “transient target” for axon sprouting and play an important role in the regulation of axo-glial interactions [8]. However, it is indicated that Sc always takes much time to proliferate and migrate into the terminal end of lesioned nerves [9]. As the functional maintenance of peripheral nerves is crucially dependent on proper signaling between Sc and axons [10], detail investigating the signal pathway involved in axo-glial interaction will not only help us to better understand the molecular mechanisms of neuro-regeneration, but also provides important insights into the clinical design of therapeutic agents that facilitate Sc migration following PNI.

Neuregulin-1 (NRG1) is one of a family of growth factors essential for the survival, proliferation, differentiation, and migration of both neurons and glia cells during development [11], [12]. Through binding to erbB2/3 receptors, NRG1 could activate numerous signal transduction pathways that regulate multiple aspects of Sc activity [13], [14]. At least two major forms of NRG1 (α and β) differing in the sequence of approximately 6∼8 amino acids have been described [15]. The β form of NRG1 (NRG ß1) is by far the most widely studied and has been reported to be more effective than α one through high binding affinity to erbB receptors [16]. *In vitro* evidences have demonstrated that NRG ß1 is a potent mitogen for developing Scs and could promote the motility of a number of cell types [17]–[19]. Pharmacological studies also reported that ablation of NRG1 would slow the progress of nerve regeneration and impair the functional recovery following nerve injury [20]. It is indicated that endogenous NRG1 may act as a chemoattractant to Scs and play an important role in the regulation of Sc migration after PNI [20]. With regard to this viewpoint, endogenous activation or exogenous applications of NRG1 would thus serve as a practical way to speed the Sc migration and facilitate the nerve regeneration under severe neuronal damage.

However, although the functional role of NRG1 in the regulation of Sc activity during development has been well documented, the potential effect of NRG1 and its downstream pathway engaged in the modulation of Sc migration through adulthood has not yet been reported. Moreover, whether exogenous treatment of NRG1 would significantly improve the nerve regeneration by greatly speeding the Sc migration following PNI is still remained to be explored. Considering focal adhesion kinase (FAK) is an essential molecule participated in the regulation of cell migration via NRG1 mediated erbB2/3 activation [21], the present study is firstly aimed to examine the potential expression of NRG1-erbB-FAK signaling in the promotion of mature Sc migration by the *in vitro* analysis. Secondly, in order to test the *in vivo* effects of NRG1 on facilitating the nerve regeneration following PNI, the degree of muscle re-innervation as well as the extents of Sc migration was assessed at different time points under the lesion model of chronic end-to-side neurorrhaphy (ESN). As ESN has previously been reported to take much time to attain successful nerve regeneration than that of general neurectomy [22], this model was thus served as a good paradigm for providing enough time courses for us to evaluate the speeding functions of NRG1 in the regenerative process following traumatic nerve injury.

## Materials and Methods

### RSC96 Cell Culture

RSC96 cells were purchased from the Bio-resource Collection and Research Center (BCRC, Hsinchu, Taiwan) and were cultured in Dulbecco’s modified Eagle’s medium (DMEM) (Invitrogen, Carlsbad, CA, USA) supplemented with 10% fetal bovine serum (FBS), 4 mM L-glutamate, 1.5 g/L sodium bicarbonate and 4.5 g/L glucose in humidified atmosphere of 5% CO_2_ and 95% air. After incubation, the cells were harvested and extracted for further analysis.

### Cell Adhesion Assay

Cell adhesion assay were performed according to the previous study [23]. Ninety-six well plates (Nalge Nunc, Rochester, NY, USA) were first blocked with 1% (wt/vol) bovine serum albumin (BSA) at 37°C for 2 hours. Cultured RSC96 Cells (2×10^4^) were then trypsinized, washed with DMEM, and re-suspended in 100 µL serum-free DMEM with NRG β1 treatment (PeproTech, Rocky Hill, NJ, USA) at the concentration of 10 nM. Following that, cells were allowed to attach for 30 min. at 37°C in a humidified incubator of 5% CO_2_. Nonspecific adherent cells were removed by washing the well with phosphate buffer saline (PBS). The number of specific adherent cell (with or without NRG β1 treatment) was then counted manually with an inverted microscope.

### Cell Spreading Assay

For cell spreading analysis, twenty-four well plates (Nalge Nunc, Rochester, NY, USA) were first coated with poly-L-lysine (PLL, 1 µg/mL) (Sigma-Aldrich, St. Louis, MO, USA) at 4°C. Nonspecific binding was blocked by 1% (wt/vol) BSA for 1 hour at 37°C. Cultured RSC96 cells were then seeded at a density of 5×10^4^ cells per well in 1 mL DMEM and 10% FBS. NRG β1 at the concentration of 10 nM was added and the cells were allowed to spread for 17 hours at 37°C. Following that, cells were washed with PBS three times, and then fixed in 4% paraformaldehyde solution. Six separate fields per well were photomicrographed with an inverted microscope. Cell spreading was characterized by the formation of a clearly defined cytoplasmic halo around the cell nucleus and spindle process as described previously [14]. RSC96 cells demonstrating this phenomenon were counted and the percentage of the total number of RSC96 cells per field was calculated. Results from six fields were then averaged to get the mean percentage of each well.

### Cell Migration Assay

Cultured RSC96 cells (3×10^4^) re-suspending in serum-free DMEM were added to the top well of each migration (Boyden) chamber with the pore membrane size of 8 µm (Transwell, Corning Life Sciences, Acton, MA, USA). Cell migration was induced by serum-free DMEM with or without NRG β1 treatment (10 nM) in a CO_2_ incubator at 37°C for 24 hours. After that, the membrane was removed and the cells on the top side of the membrane were wiped off. The remaining migrating cells on the membrane were then fixed with 100% methanol and subsequently stained with crystal violet for 3 min. Photomicrographs were taken under light microscopy (Olympus CX31RTSF, Japan) and the number of migrating cells from six random fields per chamber was counted.

### ErbB2/3 Immunofluorescence

Cultured RSC96 cells grown on glass coverslips were first fixed for 30 min. with 4% paraformaldehyde. After blocking with 1% BSA, 0.3% (vol/vol) Triton X-100 and 1% normal goat serum for 30 min, the cells were incubated with blocking buffer containing primary antibodies against active form of erbB2 and erbB3 (dilution 1∶100, Santa Cruz, CA, USA) at 4°C overnight. After washing with PBS thoroughly, cells were incubated with cy3-conjugated secondary antibody (Jackson Immunoresearch, West Grove, PA, USA) for 1 hour at room temperature. Following that, cells were washed and mounted with ProLong Gold anti-fade reagent with DAPI nuclear (Invitrogen, Carlsbad, CA, USA). The immuno-expression of erbB2 and erbB3 were photomicrographed with the Leica TCS SP5 spectral confocal system (Leica, Wetzlar, Germany).

### Western Blot Analysis

Cultured RSC96 cells were seeded onto PDL pre-coated plates as described previously [14]. After 2 days *in vitro*, cells were starved for 4 hours and treated with NRG β1 (10 nM) for 30 min. Following that, cells were scraped and the proteins were separated in 6% gradient SDS-PAGE. Nonspecific protein binding was stopped in blocking buffer containing 5% milk, 20 mM Tris-HCl (pH 7.6), 150 mM NaCl, and 0.1% Tween 20. For detection of erbB2, erbB3 and its downstream signaling molecules, anti-phospho-tyrosine antibody 4G10 (Upstate Biotechnology, Lake Placid, NY, USA), anti-erbB2, anti-erbB3 (Santa Cruz, CA, USA), anti-FAK (Biosources, Nivelles, Belgium), anti-phosphorylated FAK (p-FAK, Biosources, Nivelles, Belgium), and anti-β-actin (BD Pharmingen, San Jose, CA, USA) primary antibodies were applied to the nitrocellulose membranes. For detection of Schwann cells expression, anti-S-100β antibody (Sigma-Aldrich, St. Louis, MO, USA) was used. Immunoblotted membranes were then incubated with HRP-conjugated streptavidin, HRP-conjugated anti-rabbit IgG, or anti-mouse IgG (Santa Cruz, CA, USA). Immuno-signals were visualized with ECL reagents (Amersham Biosciences, Piscataway, NJ, USA). For quantification of immunoblottings, films were scanned and the intensity along with the ratio of specific p-FAK and FAK bands were quantified using the ImageJ 1.45 software (National Institute of Health, USA).

### Immunoprecipitation

In order to determine if erbB2 receptors were rapidly phosphorylated in response to NRG β1, the immunoprecipitation experiments were further processed in the current study. Briefly, cultured RSC96 cells treated with NRG β1 were stopped by the addition of ice cold lysis buffer. The lysates were then immunoprecipitated with protein A and protein G beads (1∶1; Amersham Pharmacia, Piscataway, NJ, USA) conjugated anti-erbB2 antibody. After that, the precipitated proteins were then subjected to Western blotting as described previously.

### *In vivo* PNI Model and Animal Studies

A total of twenty-four young adult male Wistar rats (200∼250 g) obtained from the Laboratory Animal Center of the Chung Shan Medical University were used. All experimental animals were equally divided into three groups (n = 8 in each). The first group was subjected to end-to-side neurorrhaphy (ESN) while the second group receiving ESN was subsequently given the NRG β1 at a concentration of 10 nM. In the third group, no surgical or drug exposure was performed to serve as the normal un-treated control. During the experimental period, all rats were exposed to an automatically regulated light-dark cycle of 12∶12 h (light on 07∶00∼19∶00 hours) at a constant room temperature of 25±1°C.

### Ethics Statement

In the care and handling of all experimental animals, the Guide for the Care and Use of Laboratory Animals (1985) as stated in the United States NIH Guidelines (NIH publication no. 86–23) were followed. All experimental procedures with surgical exposure and NRG β1 treatment were also approved by the Laboratory Animal Center Authorities of the Chung Shan Medical University (IACUC Approval No 1456).

### Surgical Procedures and NRG β1 Delivery

In the first two experimental groups, rats were deeply anesthetized with intraperitoneal injection of 7% chloral hydrate (Sigma-Aldrich, St. Louis, MO, USA) and underwent the ESN microsurgery as described previously [15]. Briefly, an incision was made along the left mid-clavicular line to expose the left brachial plexus. The ulnar (UnN) and musculocutaneous (McN) nerves were revealed. McN was then transected at the margin of the pectoralis major muscle. Following that, an epineurial window matching the size of McN was slit open on the UnN, taking care not to damage its containing axons, so that the cut end of McN could be attached to the UnN (end-to-side) with 10-0 nylon sutures. Immediately after ESN, animals in the second group were received 2 µL of NRG β1 treatment (10 nM, a dose which in a number of different assays we have found to be optimum in regulating Sc function) injecting directly into the distal end of McN. As NRG β1 has a short circulating half-life of approximately 30 min, the distal end of McN soaked with NRG β1 was enclosed by Gelfoam (Pharmacia & Upjohn, Kalamazoo, MI, USA) to maintain local concentration of NRG β1 and sustain the pharmacological effect. The functional recovery and the effects of NRG β1 on nerve regeneration were evaluated by retrograde tracing (DiI), S-100 protein expression, and compound muscle action potential recordings (CMAP) one and two months later following the nerve injury.

### Functional Measurement of Nerve Regeneration by Electrophysiological Recordings

In order to test the extent of functional recovery, the compound muscle action potentials (CMAP) generated by the repaired nerve were measured by the Viking Quest electromyogram (Nicolet Biomedical, Madison, WI, USA) one month after ESN. The CMAPs were recorded from needle electrodes placed in the biceps brachii. A stimulating strength of 0.2 Hz at 11 mA was used to map the McN as reported in our previous study [24]. At least three trials were recorded at each stimulus. Normal control responses were recorded from intact McN of similarly aged normal rats with similar paradigm. Data acquired were recorded digitally and the amplitudes of the responses analyzed.

### Statistical Analysis

All quantitative data acquired from *in vitro* and *in vivo* studies were firstly underwent the Kolmogorov-Smirnov test for analyzing the pattern of normality. Those qualified (*P*>0.1) were subsequently processed for one-way ANOVA followed by Bonferroni post hoc test. The Mann–Whitney *U*-test was used if normality or equal variance test failed. The statistical significance was considered if *P*<0.05.

## Results

### NRG β1 Activates erbB2/3 Expression on RSC96 Schwann Cells

**Figure 1:**
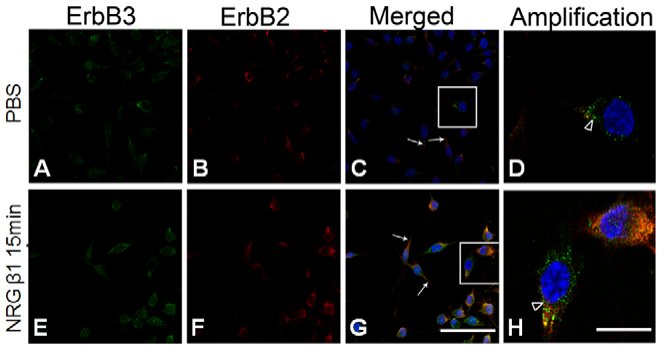
Confocal photomicrographs showing erbB2 (red) and erbB3 (green) receptor expressions in the culture RSC96 cells following phosphate buffer saline (PBS) (A–D) and neuregulin β1 (NRG β1) treatment (E–H). The cell nucleus was stained by DAPI (blue). Note that in both PBS and NRG β1 treatment groups, the erbB2 and erbB3 immunoreactivities were present in cell membrane and cell processes (arrows in C, G). However, after NRG β1 treatment (10nM) for 15 minutes, more RSC96 cells (G) with erbB2 and erbB3 co-localization (yellow) in the perinuclear area [arrowhead in (H) showing high magnification of rectangles labeled in (G)] was observed than that of PBS group (C,D). Scale bar = 20 µm in (A, B, C, E, F, G) and represents 5 µm in (D, H).

In order to evaluate whether NRG β1 would effectively activate the erbB2/3 receptor of RSC96 Schwann cells, the erbB2/3 expression was detected by immunofluorescence. The results indicated that all RSC96 cells were positively stained for erbB2 and erbB3 with or without NRG β1 treatment (Figs. 1A,B,E,F). The erbB2 and erbB3 immunoreactivities were predominantly presented in the cell membrane and cell processes (Figs. 1C,G). However, following NRG β1 treatment, both the erbB2 and erbB3 were significantly expressed throughout the full length of spindle processes (Fig. 1G, arrows) and aggregated to the perinuclear region (Fig. 1H, arrowhead) as compared to that without NRG β1 treatment.

### NRG β1 Promotes RSC96 Schwann Cells Attachment

**Figure 2:**
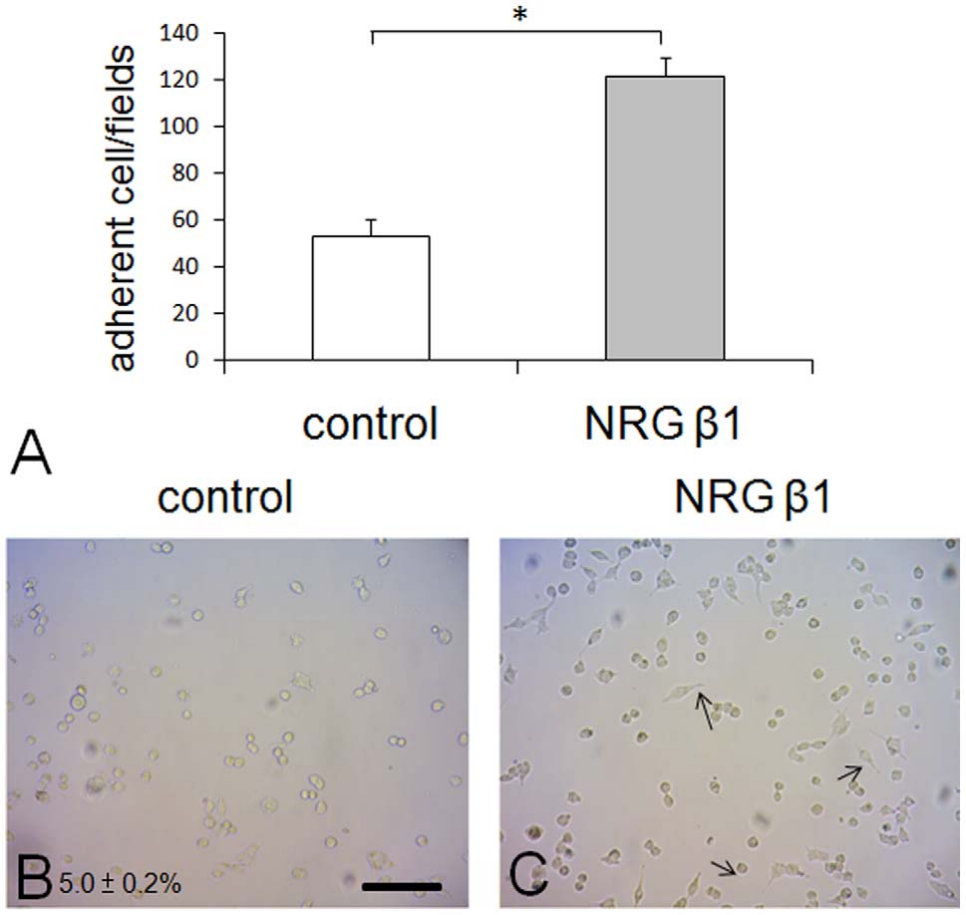
Histogram (A) and photomicrographs (B, C) showing the cell adhesion and spreading assay in culture RSC96 cells following phosphate buffer saline (PBS, control) and neuregulin β1 (NRG β1) treatment. Note that in cell adhesion assay, the number of adhered cells treated with NRG β1 was significantly larger than that of PBS control group (A). Also note that in cell spreading assay, more spreading cells with prominent spindle processes (arrows in C) were observed in NRG β1 treated group as compared to that of control ones (B). Scale bar = 40 µm. *P<0.05 as compared to that of control value.

With the purpose of investigating the potential effects of NRG β1 on cell attachment, the cell adhesion, spreading, and migration assays were performed on RSC96 Schwann cells. The results indicated that NRG β1 effectively enhanced the ability of cell attachment by significantly increasing the adherent cells to 127.2±5.0% as compared to that of control group (Fig. 2A). Subsequently cell spreading assay corresponded well with cell attachment in which NRG β1 noticeably increase the percentage of cell spreading up to nearly five-folds (24.0±2.2% in NRG β1 treated group vs. 5.0±0.2% in control group) than that of control ones (Fig. 2B).

### NRG β1 Promotes RSC96 Schwann Cells Migration

**Figure 3:**
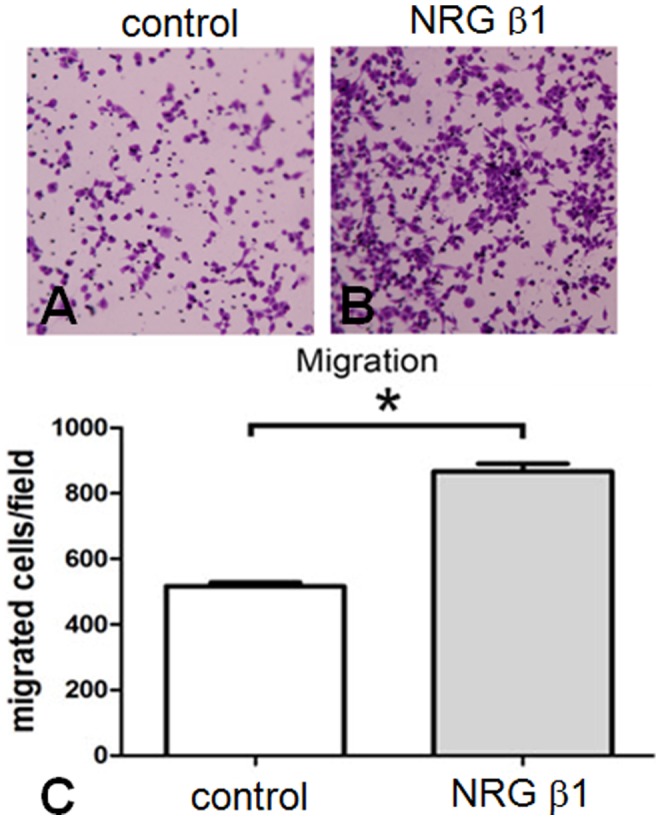
Photomicrographs (A, B) and histogram (C) showing the cell migration assay in culture RSC96 cells following phosphate buffer saline (PBS, control) and neuregulin β1 (NRG β1) treatment. The extent of cell migration was assessed by the Boyden chamber system. Note that numerous migrated cells stained with crystal violet were observed in NRG β1 group (B) as compared with that of PBS control ones (A). Also note that similar findings were detected by quantitative counting in which NRG β1 promotes more cell migration than that of PBS control group (C). *P<0.05 as compared to that of control value.

The extent of cell migration was assessed by the Boyden chamber system. In cells exposed to NRG β1 treatment (Fig. 3B), the number of migrated cells was significantly larger than that of control group (Fig. 3A). Quantitative data showed that the extent of cell migration was 800±57 cells per observed field in NRG β1 treated group as compared to that of 508±8 cells in control group (Fig. 3C).

### NRG β1 Induces Rapid Tyrosine Phosphorylation of erbB2 in RSC96 Schwann Cells

**Figure 4:**
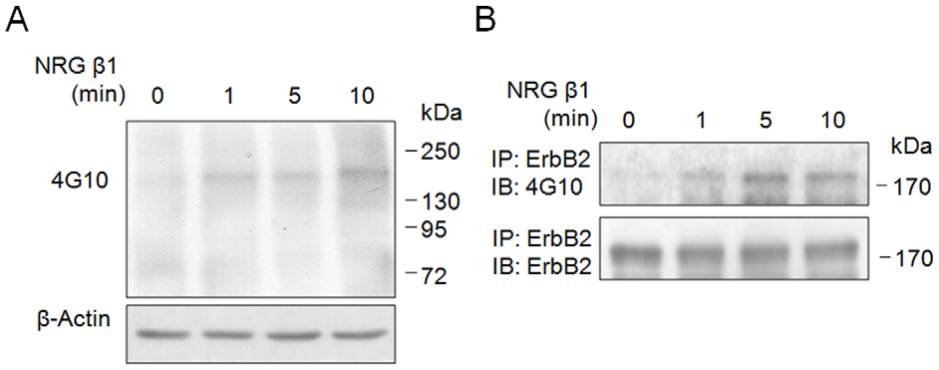
Immunoblotings showing the effects of neuregulin β1 (NRG β1) on tyrosine phosphorylation of erbB2 receptor at different time points. Note that NRG β1 significantly induces rapid tyrosine phosphorylation of membrane protein (detected by 4G10 antibody) at 185 kDa (A). The 4G10-reactive phosphorylated protein at 185 kDa was further validated to be erbB2 receptor by immunoprecipitating with anti-erbB2 antibody and followed by Western blot analysis (B). The β-actin was used as an internal loading control.

Figure 4 immunoblotting showing an increase in tyrosine phosphorylation signal of 185 kDa corresponding to erbB2 receptor identified at 1 min. following NRG β1 treatment. The NRG β1 induced tyrosine phosphorylation of whole membrane proteins was first detected by 4G10 (Fig. 4A), and then the 4G10-reactive phosphorylated protein was further confirmed to be erbB2 receptor by immunoprecipitation experiments (Fig. 4B).

### NRG β1 Stimulates RSC96 Schwann Cells Migration through erbB2/3-FAK Pathway

**Figure 5:**
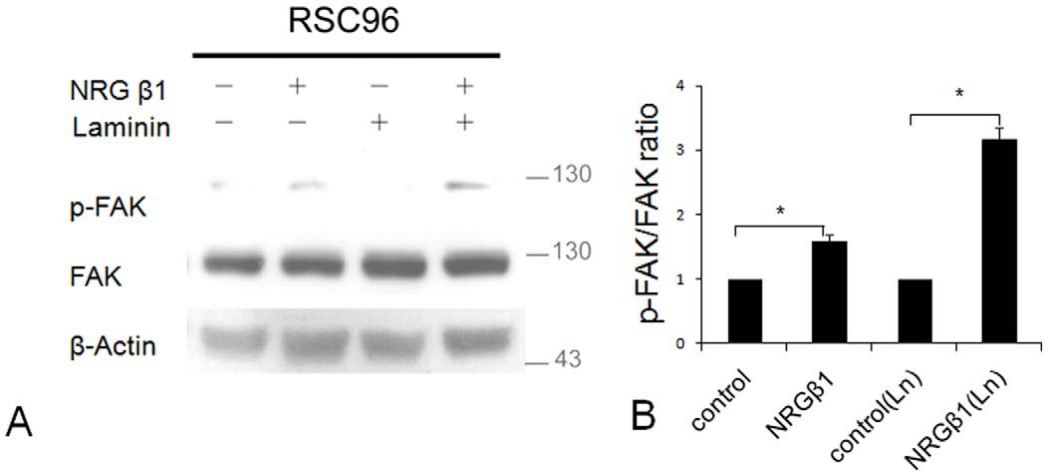
Immunoblotings (A) and histogram (B) showing the effects of neuregulin β1 (NRG β1) on focal adhesion kinase (FAK) phosphorylation in RSC96 cells seeding on poly-L-lysine (PLL) or laminin-coated plates. The physical association of erbB-FAK signaling was analyzed by the ratio expressed as phosphorylated FAK (p-FAK) over total FAK. Note that NRG β1 extensively increases erbB-FAK activation in both PLL and laminin-coated plates with the most significant effect observed in the later group. *P<0.05 as compared to that of control value.

As attempts to investigate whether the effects of NRG β1 were mediated by the intracellular erbB2/3-FAK pathway, the physical association of NRG β1 stimulation and FAK activation was assessed by the western-blot analysis. The results indicated that NRG β1 significantly induce FAK phosphorylation in cells seeding either on poly-D-lysine (PDL) or laminin-coated plates (Fig. 5A). Densitometric analysis [as expressed by the ratio measure from phosphorylated FAK (p-FAK) over total FAK] also indicated that NRG β1 effectively stimulated intracellular FAK activation with the response more efficient in cells seeding on laminin-coated environment (1.6 fold vs. 3.2 fold in PDL and laminin-coated plates, respectively, as compared to those of NRG β1-negative groups) (Fig. 5B).

### Effect of NRG β1 on Speeding the Nerve Regeneration Following ESN Injury

**Figure 6:**
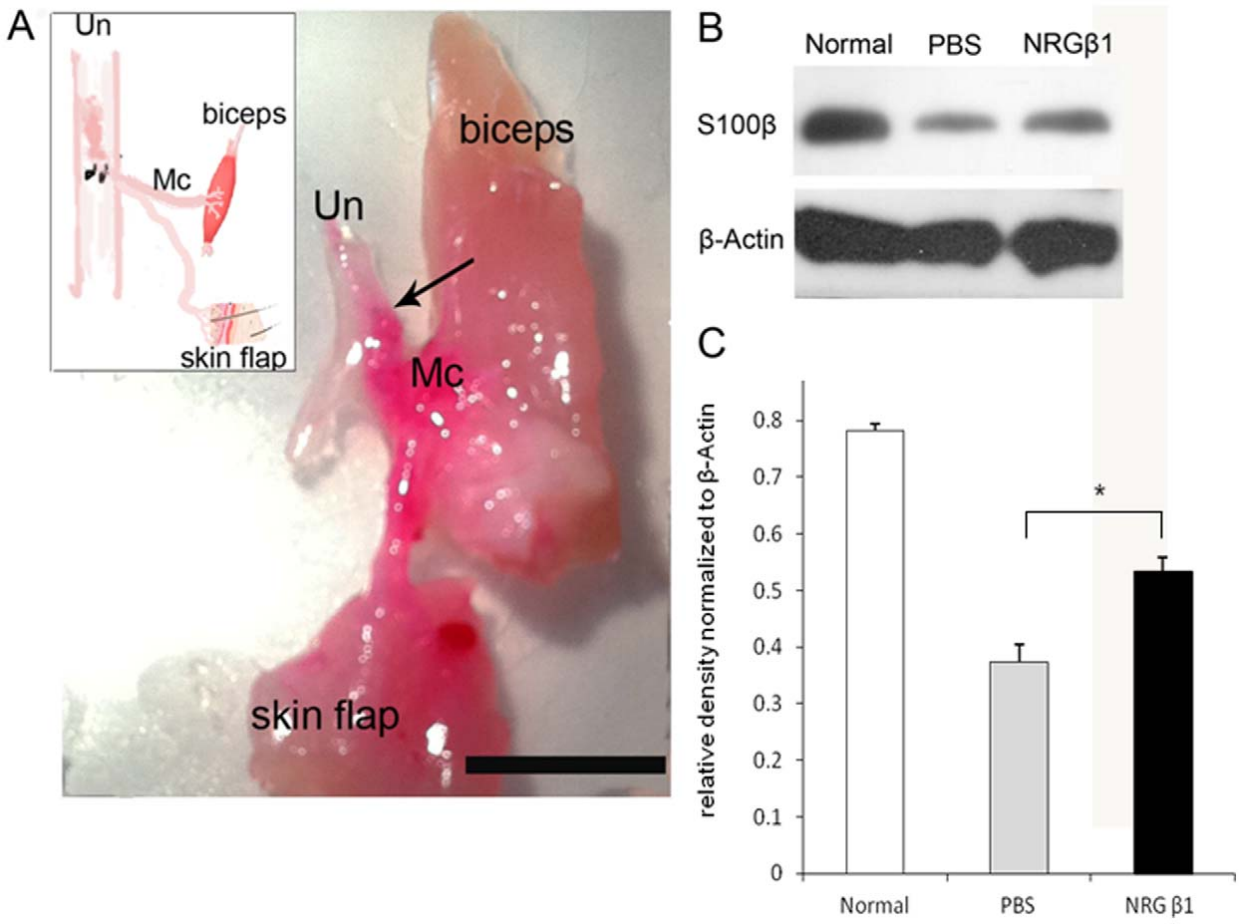
Photograph (A) and schematic diagram [inset in (A)] showing the effects of neuregulin β1 (NRG β1) on the process of nerve regeneration two months after end-to-side neurorraphy (ESN). The regenerated axons were labeled by the retrograde tracer DiI (red). Note that after NRG β1 treatment, the majority of axons from ulnar nerve (Un) successfully innervates the biceps muscle and skin flap via the side-implanted musculocutaneous nerve (Mc, arrow indicates the suture site. Scale bar = 1 cm). Also note that this good neuro-regeneration was accompanied by the higher amounts of Schwann cells migrated to the Mc following NRG β1 as compared to that of phosphate buffer saline (PBS) treated group [detected by S100β immunoblotting (B) and expressed in quantitative histogram (C)]. *P<0.05 as compared to that of PBS treated value.

**Figure 7:**
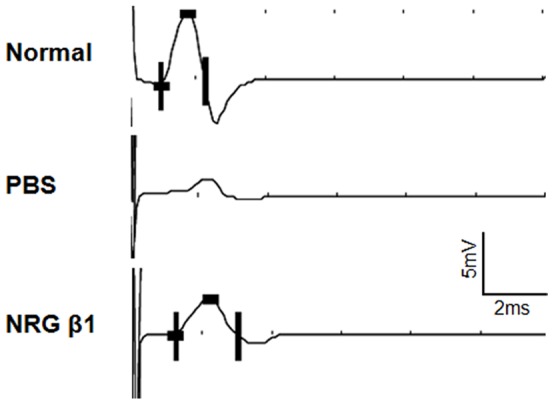
Compound muscle action potential recordings (CMAPs) of the biceps brachii muscle upon activation of the musculocutaneous nerve one month following end-to-side neurorraphy (ESN). Responses were recorded from normal rats (upper panel), phosphate buffer saline (PBS) treated rats (middle panel) and neuregulin β1 (NRG β1) treated rats (lower panel) with stimulus applied above the neurorrhaphy site. Note that NRG β1 effectively promotes nerve regeneration and functional recovery by triggering larger CMAP than that of PBS-treated ones.

In the present study, we employed the end-to-side neurorraphy (ESN) as a PNI model to detect the possible effects of NRG β1 on facilitating the processes of nerve regeneration. Behavioral test indicated that at one month following ESN, the phosphate buffer saline (PBS) treated rats could raise the affected forepaw to groom below the eyes. However, in animals treated with NRG β1, they could raise the injured forepaw to groom the ears and even to the posterior side of the ears at one and two months following ESN, respectively. The beneficial effects of NRG β1 on facilitating nerve regeneration was further validated by retrograde labeling and electrophysiological recordings in which the regenerated axons successfully re-innervated the target muscles and generated higher CMAPs (3.0±0.2 mV vs. 1.2±0.3 mV in NRG β1 and PBS treated groups, respectively) after ESN (Figs. 6A, 7). Immunoblotting analysis of Schwann cell marker (S100β) also coincided with the behavioral and neurochemical findings in which NRG β1 promoted larger amounts of Schwann cells migrating to the distal stump as compared to that of PBS-treated group (Figs. 6B,C). These results indicated that NRG β1 could facilitate nerve regeneration by speeding the Schwann cell migration to the injured site and serve as a crucial substrate for subsequent re-myelination and axonal guidance following PNI.

## Discussion

**Figure 8:**
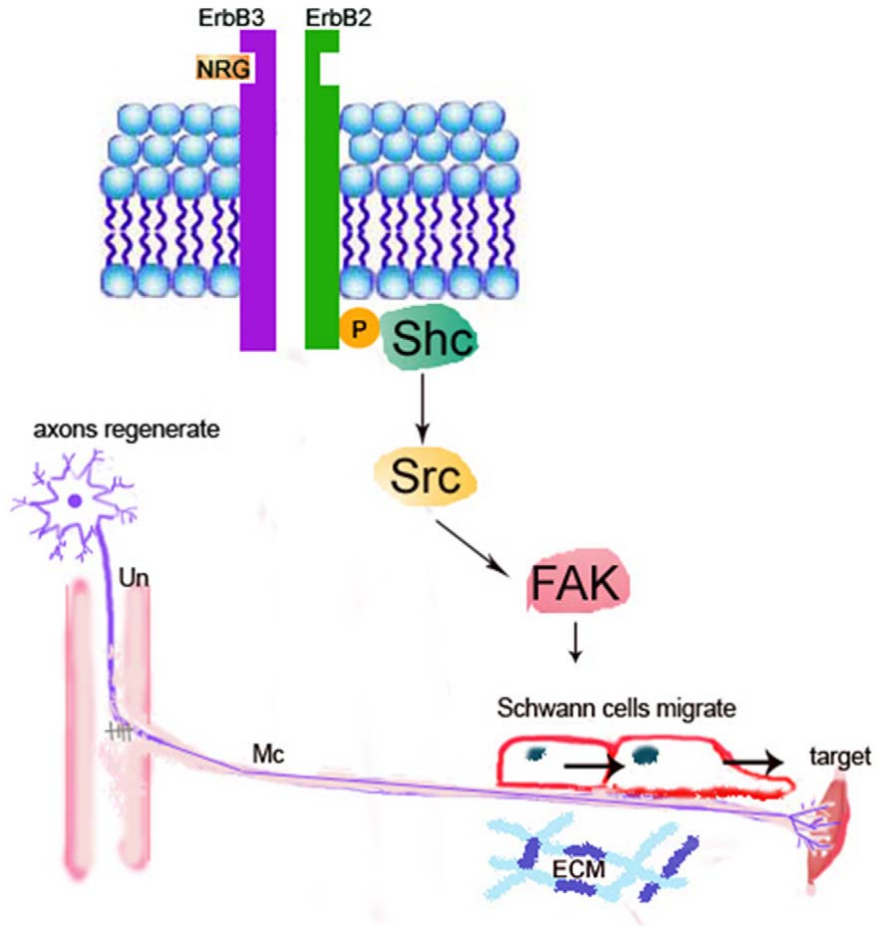
Schematic diagram showing the NRG-erbB-FAK signaling in the repairing process following peripheral nerve injury (PNI). Following PNI [as created by end-to-side neurorraphy (ESN)], NRG would activate the erbB2-FAK pathway that promotes the Schwann cells to migrate to the distal stump for successful axonal guidance and nerve regeneration. Un: ulnar nerve; Mc: musculocutaneous nerve; ECM: extracellular matrix.

The present study is the first one employing both *in vitro* and *in vivo* approaches to clear provide the functional anatomical evidence that NRG β1 could significantly facilitate nerve regeneration by speeding Sc migration following peripheral nerve injury. The advanced effects of NRG β1 was mediated by erbB2/3 receptors, which subsequently promotes FAK phosphorylation and activates the downstream signaling related to migration activity (Fig. 8). It is indicated that adequate migration of Sc is crucial for the regeneration of injured nerves [6], [7]. By producing various kinds of functional substances such as diffusible neurotrophic factors, extracellular matrix and cell adhesion molecules, Sc could provide structural stability of the regenerating axons that facilitates axo-glial interaction and promotes functional recovery [25]–[27]. During the past few years, the NRG1-erbB signaling has emerged as a key regulator of axo-glial interaction, which plays an important role in the differentiation, proliferation, maturation and migration of Sc during development [28]. A variety of evidences have demonstrated that animals lacking NRG1 or erbB2/3 would cause a complete absence of Sc precursor generation and inhibit the migration of Sc beyond dorsal root ganglion into the peripheral nerves [29]–[31]. Other than regulate the developmental progress of Sc, NRG1-erbB signaling has also been reported to contribute to the regenerative function following adult peripheral nerve injury [20]. It is indicated that exogenous treatment of NRG β1 would increase the length of regenerating axons and improve the functional outcome after sciatic nerve injury [32], [33]. Pharmacological report also documented that following facial nerve transection, NRG β1 could increase the Sc nuclei and reduce the myelin debris [34]. Our current study was thus in good agreements with these findings in which application of NRG β1 also showed an increase of migrated Sc and speeding the nerve regeneration following end-to-side neurorraphy (Figs. 6, 7). However, although NRG1 has beneficial effects on peripheral regeneration, the potential mechanism(s) of how NRG1 signaling may modulate the nerve regeneration has not been extensively explored. With regard to this viewpoint, our present study further examined the intracellular FAK expression, which has previously been shown to participate in signal transduction induced by laminin and serve as the downstream molecule engaged in Sc migration through NRG1-erbB signaling [21]. The results indicated that NRG1 significantly enhanced FAK expression in both PLL and PLL plus laminin coated slides (Fig. 5). The increased level of FAK expression following NRG1 exposure clearly implies that NRG1 and laminin could act synergistically to enhance Sc migration through the NRG-erbB-FAK pathway. To our knowledge, this study is the first one presenting both *in vivo* and in *vitro* evidences to clarify the advanced effects (and its underlying mechanism) of NRG1. By great unraveling and manipulating of such signaling events will give us new opportunity to facilitate nerve repair and promote the functional restoration following peripheral nerve injury.

In the present study, we use the RSC96 cell line to detect the degree of cell attachment, cell migration and erbB2/3 immuno-reactivities following NRG1 treatment. One might concerned that the endogenous properties of immortalized Sc is different to that of primary Sc, which is therefore not suitable to use as a model for cellular analysis. As RSC96 cell line is commonly utilized in the analyses of cell mobility [23], [35] and is well-characterized in their enriched expression of Sc related protein when compared to that of primary Sc [36], the RSC96 cell line was thus serve as a good material to evaluate the functional effects of NRG1 on Sc protein expression and cellular activities.

Another important issue to be addressed is that the erbB2/3 expression would localize to the perinuclear area following NRG1 treatment (Fig. 1). Similar finding has also been reported by Adilakshmi et al. in which a nuclear-localized form of erbB3 was observed under the addiction of exogenous NRG1 [37]. It is indicated that the nuclear expression of erbB was associated with the genome regions including promoters that regulate the expression of proteins involved in several Sc functions [37]. Based on this viewpoint, it is possible to suggest that NRG1 stimulation could also drive the expression of an alternatively spliced transcript of the erbB gene in Sc, which subsequently resulting in transcriptional regulation of numerous genes participated in the migration and other Sc activities.

In summary, the present study has provided the first functional anatomical evidence that NRG1 could significantly facilitate the nerve regeneration by speeding Sc migration through NRG1-erbB-FAK pathway. Although the positive effects of NRG1 on nerve regeneration may not restricted to enhance Sc migration, this work has still shed light on the functional significance of this axo-glial signaling, which may has great help for us to develop novel strategy to combat the PNI-induced neuronal disability.
